# Prediction of grip and key pinch strength in 978 healthy subjects

**DOI:** 10.1186/1471-2474-11-94

**Published:** 2010-05-19

**Authors:** Felix Angst, Susann Drerup, Stephan Werle, Daniel B Herren, Beat R Simmen, Jörg Goldhahn

**Affiliations:** 1 Department of Upper Extremity and Hand Surgery, Schulthess Klinik, Lengghalde 2, 8008 Zurich, Switzerland; 2 Rehaclinic Zurzach, Quellenstrasse, 5330 Bad Zurzach, Switzerland; 3 Institute for Biomechanics of Swiss Federal Institute of Technology (ETH), 8090 Zurich, Switzerland

## Abstract

**Background:**

Hand strength is an important independent surrogate parameter to assess outcome and risk of morbidity and mortality. This study aimed to determine the predictive power of cofactors and to predict population-based normative grip and pinch strength.

**Methods:**

A representative population survey was used as the basis for prediction analyses (n = 978). Bivariate relationships between grip/pinch strengths of the dominate hand were explored by means of all relevant mathematical functions to maximize prediction. The resulting best functions were combined into a multivariate regression.

**Results:**

Polynoms (up to the third degree) were the best predictive functions. On the bivariate level, height was best correlated to grip (46.2% explained variance) and pinch strength (37.7% explained variance) in a linear relationship, followed by sex, age, weight, and occupational demand on the hand. Multivariate regression provided predicted values close to the empirical ones explaining 76.6% of the variance for grip strength and 67.7% for pinch strength.

**Conclusion:**

The five easy-to-measure cofactors sex, age, body height, categorized occupational demand on the hand, and body weight provide a highly accurate prediction of normative grip and pinch strength.

## Background

The hand represents the most sophisticated and differentiated musculoskeletal tool in the human being, demanding the largest capacity of the nervous system in relation to its size. Full function and adequate strength of the hand are preconditions for dealing with the demands of daily life. Hand strength has been identified as an important factor predicting not only disability in musculoskeletal diseases such as rheumatoid arthritis [1], but also bone mineral density [2,3], and the likelihood of falls and fractures in osteoporosis [4,5]. It even predicts complications and general morbidity after surgical interventions [6], general disability and future outcome in older age [7-9], economic consequences of diseases [10] as well as cause-specific and overall mortality in elderly people [5,11-13]. Arteriosclerosis is the most frequent cause of morbidity and mortality and grip strength is one of the strongest predictors of its consequences, e.g. myocardial infarction or stroke and post-event recovery [12,14,15]. In contrast, grip strength is almost completely independent from depression and return to work [16,17].

In addition to its predictive value grip strength and key pinch strength are considered to be "objective" outcome parameters and are used to quantify outcome after orthopaedic interventions [18]. Many investigators normalize measured values such as percentage of the contralateral, non-affected side. However, when the contralateral side is also compromised by the underlying disease or its treatment, other benchmarks are required. Although numerous publications provide normative data for grip strength and key pinch strength, it would be helpful to predict the expected hand strength values for an individual based on easily measured factors [19].

Few studies examined predictors of hand strength itself. Strong predictors are sex, age, body height and mid-forearm circumference [20,21]. Weaker predictors are body weight and hand size measures [21]. Grip strength has often been taken as surrogate for overall strength but this should be done with caution since correlation of the two strength measures is high in many but also low in few settings [22]. Grip strength has a substantial north-south gradient from 24.2 kg in Denmark to 14.2 kg in Calabria in men [23,24]. Age-and gender-specific grip and key pinch strengths have recently been evaluated in a large population-based field study in Switzerland [25]. We used the data from that study to determine the predictive impact of various cofactors.

This study aimed to quantify the predictive power of easily assessable demographic and/or anatomical factors such as sex, age, occupational demands on the hand, body height, and body weight on grip and pinch strength. The second aim was to predict grip and pinch strength by a regression model of these factors.

## Methods

### Data collection

Subjects were selected at convenience at shopping centres, malls, secondary schools, senior sports groups, and senior residences (to find also immobile persons) in the German speaking part of Switzerland "to realize a random approach" [25]. Approval for the data collection of that study was obtained from the local ethics committee [25]. Each person had to undergo grip strength testing using a Jamar dynamometer and key pinch testing using a pinch gauge. Grip and pinch testing was performed in a standardized way according to the recommendations of the American Society of Hand Therapists as described in detail in [25]. Especially, the Jamar dynamometer was calibrated before the first measurement and was applied in the second handle position.

The average of three measurements per person was recorded and entered into a database together with the assessment of cofactors that had been identified as confounders in the clinical literature. They were: sex, age, body height, body weight, and demands on the hand due to occupational activity (classified into six categories: beyond sedentary, sedentary, light, medium, heavy, very heavy) as set out in the directory of occupational titles [26]. Dominant handedness was also determined by a standardized questionnaire [25].

All to the present analysis underlying data are described in detail in the descriptive original report [25]. In 2006 and 2007, data were obtained for 1023 persons. 4.4% were classified as ambidextrous and excluded from the analysis. The remaining 978 persons provided the data for the grip and pinch strength tables of the population survey and for the present study. There were 496 men and 482 women, of whom 88.3% were right-handed. The dominant hand was the right hand for 87.8% of the men and 88.8% of the women. Age ranged between 18 and 96 years and was classified into 5-year groups. Strength data were determined by n = 28 to 46 male subjects and n = 26 to 42 female subjects within the sex and age-group strata. Body height was on average 175.1 cm (standard deviation 7.1 cm) for men and 163.7 cm (6.7 cm) for women. The corresponding values for body weight were 78.0 kg (11.8 kg) for men and 64.1 kg (12.3 kg) for women. Frequency data within the occupational levels were as follows: beyond sedentary work: men 10.9% / women 10.1%, sedentary work: 48.1% / 41.4%, light work: 15.7% / 21.7%, medium work: 20.3% / 26.2%, heavy work: 5.0% / 0.6%, very heavy work: 0.0% / 0.0%. Detailed means and standard deviations of grip and pinch strength stratified by sex and 5-year age groups are shown in table 1, an extract of the tables of the original report.

**Table 1:** Descriptive data of normal grip and pinch strength (kg), dominant hand (n = 978)

Age	Grip				Pinch			
	M		F		M		F	
	m	s	m	s	m	s	m	s
**18-19**	51.2	6.6	32.0	4.8	9.5	1.8	6.9	1.2
**20-24**	53.9	8.7	33.4	5.4	9.8	1.4	6.5	1.3
**25-29**	53.0	7.5	34.3	5.7	10.1	1.4	6.8	0.9
**30-34**	55.0	7.1	33.8	5.9	9.9	1.5	6.9	1.2
**35-39**	55.9	7.9	35.8	6.7	10.4	1.5	7.1	1.4
**40-44**	54.2	8.1	36.0	6.0	10.3	1.5	7.2	1.0
**45-49**	51.8	8.3	34.1	5.3	9.8	1.7	7.1	1.3
**50-54**	50.8	9.1	33.7	4.5	9.7	1.5	6.9	10
**55-59**	53.6	8.6	31.9	4.9	10.3	1.5	6.8	1.4
**60-64**	47.9	6.4	28.7	5.5	9.8	1.5	6.7	1.4
**65-69**	43.0	6.8	29.5	3.6	8.7	1.5	6.3	1.1
**70-74**	41.7	8.9	26.4	6.8	8.3	1.9	5.7	1.6
**75-79**	36.8	9.7	25.0	4.5	8.2	2.4	5.1	1.2
**80-84**	30.7	9.1	19.2	5.2	6.4	2.1	4.3	1.3
**≥85**	22.4	6.2	16.9	4.8	5.4	1.8	3.1	1.3

Age in years. n = number of subjects. M = male, F = female. m = arithmetic mean, s = standard deviation.

### Analysis

Grip and pinch strength in kg were predicted as independent variables for the dominant hand. First, bivariate curve adaptations were calculated using linear, quadratic, cubic, inverse, composite (inverse and polynom together), exponential, logarithmic, logistic, and s-shaped function characteristics of the dependent variable to find the optimal curve adaptation for each single cofactor explaining maximal variance of the dependent strength variable. The body mass index, BMI = weight/height^2 ^was also taken into account by height and weight and by modeling all possibilities of linear, quadratic and inverse terms. In principle, every bivariate characteristic can be approximated by a polynom, y = a_0_+a_1_x+a_2_x^2^+...+a_n_x^n^, the higher the degree n the more precise the approximation [27].

In a second step, all single optimal functions were combined together into a stepwise multivariate multifunctional regression to maximize explained variance. Regression coefficients, level of significance for predictive power, bivariate and partial correlations were determined. Addition and omission of one regression term had to significantly change the r square (=explained variance) of the regression model for stepwise inclusion as being tested by the F-test [28,29]. All possible order of the variables were tested in adding / omitting to find the optimal model.

The coding was 0 = f, 1 = m for sex, age in years, height in cm, weight in kg, 0 = beyond sedentary, 1 = sedentary, 2 = light, 3 = medium, 4 = heavy, 5 = very heavy for the variable of occupational demands. All analyses were performed using the statistical software package SPSS 17.0 for Windows^® ^(SPSS Inc., Chicago, IL, USA).

## Results

### Grip strength

The results of the bivariate and the mulivariate regressions of the cofactors with grip strength are shown in Table 2. All optimal models were polynoms, i.e. other functions like inverse or logarithmic did not explain more variance and did not predict grip strength better than a polynom. The optimal polynom was linear (i.e. grip strength = a_0 _+ a_1_covariate) for sex, age, and height, quadratic (a_0 _+ a_1_covariate + a_2_covariate^2^) for age and weight, and cubic for occupational demand (a_0 _+ a_1_covariate + a_2_covariate^2 ^+ a_3_covariate^3^). Height (46.2% explained variance) had the highest bivariate predictive power, followed by sex, age, occupation and weight (see bivariate explained variance in Table 2).

**Table 2:** Bivariate relationship and multivariate regression data of normal grip strength dominant (multivariate explained variance: 76.6%) (n = 978)

Covariates	Bivariate		Multivariate			
	Correlation^4^	Explained variance^5^	Partial correlation	Explained variance^6^	Regression coefficient	Coefficient's significance
Constant	-	-	-	-	-28.148	<0.001
Sex^1^	0.635	40.3%	0.576	33.2%	12.500	<0.001
Age^1^	-0.460	29.6%	0.187	3.5%	0.372	<0.001
Age^2^	-0.506	-	-0.263	6.9%	-0.005	<0.001
Height^1^	0.680	46.2%	0.272	7.4%	0.304	<0.001
Weight^1^	0.460	21.7%	-0.022	<0.1%	-0.083	0.502
Weight^2^	0.446	-	0.041	0.2%	0.001	0.198
Occupation^1^	0.377	24.4%	0.254	6.5%	12.293	<0.001
Occupation^2^	0.307	-	-0.225	5.1%	-5.865	<0.001
Occupation^3^	0.284	-	0.200	4.0%	0.897	<0.001

^1 ^linear: coefficient times variable, ^2 ^quadratic: coefficient times variable^2^, ^3 ^cubic: coefficient times variable^3^, ^4 ^Correlation between grip strength and the covariates, e.g. grip strength with age (linear relationship), ^5 ^Explained variance of the optimal bivariate model (maximal explained variance) of grip strength with the covariates, e.g. grip strength = a_0 _+ a_1_age + a_2_age^2^. ^6 ^Partial explained variance of the single regression terms within the optimal multivariate model (maximal explained variance).

All optimal bivariate model terms were combined to multivariate regression (Table 2). The multivariate partial correlation reflects the power of each covariate's term to predict grip strength. Sex was the strongest multivariate term and explained 33.2% of the variance of grip strength. All other terms were similarly predictive and substantially weaker, i.e. height explained only 7.4% of the variance in the multivariate model. Weight played no predictive role. This means that all other covariates explained the variance of weight in the multivariate model. Leaving weight out of the regression reduces the explained variance from 76.6% to 76.1%. Using the coefficients of the multivariate regression (second to last column of Table 2) now makes it possible to predict grip strength by using an equation:

For example, a 47-year old man 171 cm in height, weighing 72 kg and doing light work has an average grip strength of 50.3 kg in his dominant hand. The table of empirical values gives 51.8 kg, resulting in an error of 2.9% [25]. If weight is excluded from the regression analysis, the result will be 51.1 kg. The nondominant hand has an empirical value of 50.0 kg [25].

### Pinch strength

The optimal bivariate models were the same as those for grip strength, i.e. they were all polynoms: linear for sex and height, quadratic for age and weight, and cubic for occupation. The results of the bivariate and the mulivariate regressions of the cofactors for pinch strength are shown in Table 3. The predictive power was highest for height (37.7% explained variance), followed by sex, age, weight, and occupation for the bivariate models.

**Table 3:** Bivariate relationship and multivariate regression data of normal pinch strength dominant (multivariate explained variance: 67.7%) (n = 978)

Covariates	Bivariate		Multivariate			
	Correlation^4^	Explained variance^5^	Partial correlation	Explained variance^6^	Regression coefficient	Coefficient's significance
Constant	-	-	-	-	-2.637	0.066
Sex^1^	0.597	35.7%	0.492	24.2%	2.138	<0.001
Age^1^	-0.389	25.1%	0.187	3.5%	0.079	<0.001
Age^2^	-0.441	-	-0.237	5.6%	-0.001	<0.001
Height^1^	0.614	37.7%	0.146	2.1%	0.034	<0.001
Weight^1^	0.482	23.8%	0.008	<0.1%	0.007	0.796
Weight^2^	0.470	-	0.024	0.1%	0.001	0.456
Occupation^1^	0.354	22.9%	0.255	6.5%	2.641	<0.001
Occupation^2^	0.287	-	-0.230	5.3%	-1.282	<0.001
Occupation^3^	0.265	-	0.203	4.1%	0.195	<0.001

^1 ^linear: coefficient times variable, ^2 ^quadratic: coefficient times variable^2^, ^3 ^cubic: coefficient times variable^3^, ^4 ^Correlation between pinch strength and the covariates, e.g. pinch strength with age (linear relationship), ^5 ^Explained variance of the optimal bivariate model (maximal explained variance) of pinch strength with the covariates, e.g. pinch strength = a_0 _+ a_1_age + a_2_age^2^. ^6 ^Partial explained variance of the single regression terms within the optimal multivariate model (maximal explained variance).

The multivariate regression revealed sex to be the most predictive variable (24.2% explained variance) whereas all other covariates were of limited predictive power, especially weight. The regression equation is (Table 3):

For the subject described above, this results in an expected dominant pinch strength of 11.1 kg. The measured value of 9.8 kg corresponds to an error of 13.3% for the dominant hand. 9.2 kg are reported for the nondominant hand [25]. Leaving weight out of the analysis would result in 8.5 kg.

## Discussion

We quantified the predictive impact and predicted normative dominant grip and pinch strength by sex, age, body height and weight, and occupational demands on the hand based on a representative population survey [25]. The overall predictive power of these cofactors combined was very high - higher for grip than pinch strength (76.6% versus 67.7% multivariate explained variance). This was consistently true for the predictive power of the covariates in the bivariate and multivariate relationships.

The square roots of the explained variances mean that the data of the multivariate regression correlate to the empirically measured ones with values of 0.88 for grip strength and 0.82 for pinch strength [29]. For both strength parameters, polynominal equations were the best predictive functions with a maximal degree of 3 (for occupation). In the bivariate functions, height was the best and weight the worst predictor. In the multivariate approximation, sex was the most important predictor, and weight had virtually no predictive impact. The quadratic term of age approximated to the empiric curves much closer than the linear regression and reduced the high variance observed in older age, which was discussed as a possible weakness of the population survey [25].

The multivariate model provided a valid prediction of grip strength (error 2.9%) but predicted pinch strength was less close to the measured one (error 13.3%) as shown by the examples. However, the empirical data in the tables were only mean values stratified by sex and 5-year age group and left out the cofactors body height, weight, and occupational demands on the hand [25]. Unequal distribution of important confounders such as occupational demands on the dominant hand may cause bias of the explicit data in the tables. For this reason and given the fact that predictive power of the multivariate models was high, it is possible that multivariate equations provide a better estimate of grip and pinch strength for a specific person than the empirically measured data. In addition, age taken as quasi-continuous variable into the regression will provide more precise normative data than obtained by means of strength out of age-grouped tables.

The difference in the grip and pinch strengths measured in a person suffering from a somatic (but not mental) disorder compared to the normative value may be used to predict various prognostic outcomes and risks as indicated in the wider literature. Examples are morbidity and mortality of rheumatological affections, vascular diseases, and general predictions as listed in the introduction [1-15]. Furthermore, hand/grip strength measurement is an easily performed "quick bedside test" [6]. Comparing clinical data to normative values allows the assessor to qualify (on average) whether the patient is at an elevated risk or not but few studies provided clinically feasible quantification of that risk. For example, the increment of 5 kg grip strength was associated with an average decrease of 10-15% in overall mortality after adjustment for confounders [11]. On the other hand, it is not possible to give a life expectancy in remaining years for a measured grip strength of 20 kg or 30 kg when 40 kg is expected by the norm. This raises important issues for future research.

One of the strengths of the study is the large representative sample providing valid normative data for the German speaking part of Switzerland and which can be expected to be valid for other European countries and the USA. Predictive power has been proven in various countries and cultures [1-15,20,21]. The regression formula can easily be programmed and allows quick determination of the norm for a specific person. For example, grip strength in Excel: fill in sex (0 = f, 1 = m) into field A1, age in years into A2, height in cm into A3, weigth in kg into A4, occupation (0 = beyond sedentary, 1 = sedentary, 2 = light, 3 = medium, 4 = heavy, 5 = very heavy) into A5, and program the formula of grip strength into A6: -28.148+12.5*A1+0.372*A2-0.005*A2*A2+0.304*A3-0.083*A4+0.001*A4*A4+12.293*A5-5.865*A5*A5+0.897*A5*A5*A5. Get the result of grip strength in kg by "enter".

The limitations of the study are that the data may not be transferable to countries or populations with different socioeconomic conditions, especially to populations with high proportions of craftsmen. However, our grip strength data (see table 1) were comparable to those pooled by a meta-analysis of 12 studies of which 8 were conducted in the USA [19]. The prevalence of high demands on the hand due to occupational activity was low in our study, especially, there were no subjects reporting very high demands. Realistically, the prediction and regression data are only representative and valid for population-based normative values and not for disabled patients. The selection procedure of the volunteers was not really random. Furthermore, it was not possible to quantify health risks on the basis of the present data as discussed above. Finally, we did not further examine potentially predictive cofactors such as sporting or leisure activity level or comorbidities, e.g. smoking. However, there remained little space to explain additional variance since the regression models provided a good fit.

## Conclusions

The five easy-to-measure cofactors sex, age, body height, categorized occupational demand on the hand, and body weight provide a highly accurate prediction of normative grip and pinch strength.

## Competing interests

The authors declare that they have no competing interests.

## Authors' contributions

All authors commented on the draft and the interpretation of the findings, helped to write and approved the final manuscript. FA was responsible for all parts of the work of the study, especially for the design, the analysis and the interpretation of the data, and wrote the original manuscript. SD and SW were responsible for the data acquisition and helped in the analysis of the data. DH helped in the interpretation of the data. BS and JG were responsible for the conception and the resources for the study.

## Pre-publication history

The pre-publication history for this paper can be accessed here:

http://www.biomedcentral.com/1471-2474/11/94/prepub
